# Microbial Diversity and Community Structure of Sulfate-Reducing and Sulfur-Oxidizing Bacteria in Sediment Cores from the East China Sea

**DOI:** 10.3389/fmicb.2017.02133

**Published:** 2017-11-07

**Authors:** Yu Zhang, Xungong Wang, Yu Zhen, Tiezhu Mi, Hui He, Zhigang Yu

**Affiliations:** ^1^College of Environmental Science and Engineering, Ocean University of China, Qingdao, China; ^2^Key Laboratory of Marine Environment and Ecology, Ministry of Education, Qingdao, China; ^3^Laboratory for Marine Ecology and Environmental Science, Qingdao National Laboratory for Marine Science and Technology, Qingdao, China; ^4^College of Marine Life Science, Ocean University of China, Qingdao, China; ^5^Key Laboratory of Marine Chemical Theory and Technology, Ministry of Education, Qingdao, China

**Keywords:** sulfate-reducing bacteria, sulfur-oxidizing bacteria, microbial community, high-throughput sequencing, East China Sea

## Abstract

Sulfate-reducing bacteria (SRB) and sulfur-oxidizing bacteria (SOB) have been studied extensively in marine sediments because of their vital roles in both sulfur and carbon cycles, but the available information regarding the highly diverse SRB and SOB communities is not comprehensive. High-throughput sequencing of functional gene amplicons provides tremendous insight into the structure and functional potential of complex microbial communities. Here, we explored the community structure, diversity, and abundance of SRB and SOB simultaneously through 16S rRNA, *dsrB* and *soxB* gene high-throughput sequencing and quantitative PCR analyses of core samples from the East China Sea. Overall, high-throughput sequencing of the *dsrB* and *soxB* genes achieved almost complete coverage (>99%) and revealed the high diversity, richness, and operational taxonomic unit (OTU) numbers of the SRB and SOB communities, which suggest the existence of an active sulfur cycle in the study area. Further analysis demonstrated that rare species make vital contributions to the high richness, diversity, and OTU numbers obtained. Depth-based distributions of the *dsrB, soxB*, and 16S rRNA gene abundances indicated that the SRB abundance might be more sensitive to the sedimentary dynamic environment than those of total bacteria and SOB. In addition, the results of unweighted pair group method with arithmetic mean (UPGMA) clustering analysis and redundancy analysis revealed that environmental parameters, such as depth and dissolved inorganic nitrogen concentrations, and the sedimentary dynamic environment, which differed between the two sampling stations, can significantly influence the community structures of total bacteria, SRB, and SOB. This study provided further comprehensive information regarding the characteristics of SRB and SOB communities.

## Introduction

Sulfur cycling, one of the key biological processes in marine sediments, is dominated by sulfate-reducing bacteria (SRB) and sulfur-oxidizing bacteria (SOB). Dissimilatory sulfate reduction, which is mediated by SRB, is considered the main process in the biomineralization of organic matter in marine sediments and might account for up to 50% of organic matter mineralization in most continental shelf sediments (Jørgensen, 1982). In addition, as much as 12–29% of the organic carbon flux on the ocean seafloor is channeled through sulfate reduction (Bowles et al., 2014). Interestingly, 80–95% of the massive amount of hydrogen sulfide formed through sulfate reduction is recycled within sediments and gradually oxidized back to sulfate (Jørgensen and Nelson, 2004). Thus, SRB and SOB control the key processes of organic matter degradation and the biogeochemical cycling of sulfur and carbon. Studying the community structure and diversity of SRB and SOB is important for revealing the roles of these bacteria in the biogeochemical cycles of carbon and sulfur and for providing insight into the biological factors driving the marine sulfur cycle.

SRB and SOB show high diversity, including both phylogenetic and metabolic diversity (Ghosh and Dam, 2009; Müller et al., 2014). To explore the environmental abundance and diversity of SRB and SOB, genes encoding key enzymes in the sulfate reduction and sulfur oxidation biochemical pathways have been used as molecular markers. For example, the dissimilatory sulfite reductase gene *dsrAB*, which encodes a key enzyme that catalyzes the last, essential step in the dissimilatory sulfate reduction pathway, has been frequently employed as a functional gene in studies of SRB in various environments (Foti et al., 2007; Pester et al., 2012; He et al., 2015). The known SOB have been demonstrated to use different enzymes, pathways, and electron transport and energy conservation mechanisms for the oxidation of sulfide. The sulfur-oxidizing (Sox) (Meyer et al., 2007), reverse dissimilatory sulfite reductase (Loy et al., 2009), adenosine-5-phosphosulfate reductase (Meyer and Kuever, 2007), and sulfide quinone oxidoreductase enzyme systems (Pham et al., 2008) play vital roles in sulfide oxidation, and among these, the Sox multi-enzyme system is considered a fundamental and primordial molecular mechanism for sulfur oxidation (Ghosh and Dam, 2009) that is widespread among the known SOB (Meyer et al., 2007). Moreover, the *soxB* gene, which encodes the SoxB subunit of the Sox enzyme system, has been widely employed to characterize the abundance and diversity of SOB in various environments (Kojima et al., 2014; Thomas et al., 2014; Tourna et al., 2014).

The diversity and community structure of SRB and SOB have, to date, been studied using denaturing gradient gel electrophoresis (DGGE) (Varon-Lopez et al., 2013; Yang et al., 2015), restriction fragment length polymorphism (Luo et al., 2011; Reed and Martiny, 2013), and clone library approaches (Yang et al., 2013; Purcell et al., 2014). However, obtaining comprehensive information about the highly diverse SRB and SOB communities, particularly SRB and SOB from the prime habitats of marine sediments, using these methods is challenging (Aoki et al., 2015; Zhang et al., 2016). High-throughput sequencing, which constitutes a powerful approach for achieving complete coverage of microbial communities, has been recently applied in the analysis of microbial community diversity and composition. For instance, high-throughput sequencing of the 16S rRNA gene revealed the presence of a number of novel bacterial groups (Zhu et al., 2013; Mahmoudi et al., 2015) and a high diversity of bacteria (Wang et al., 2015). Moreover, the community structure and diversity of functional microbes, particularly nitrogen-cycling microbes, such as ammonia-oxidizing bacteria, ammonia-oxidizing archaea and denitrifying microbes, have been investigated through high-throughput sequencing analyses of functional genes (Tago et al., 2014; Saarenheimo et al., 2015). However, only a few studies have evaluated the community structure and diversity of SRB and SOB using high-throughput sequencing (Meyer et al., 2016; Cui et al., 2017).

The East China Sea (ECS) is the largest marginal sea of the Northwest Pacific Ocean, with a vast continental area of 0.5 × 10^12^ m^2^. The Changjiang River (Yangtze River), which transports great quantities of freshwater, nutrients, organic matter, and other chemical elements into the ECS, has a marked effect on the sea (Milliman and Meade, 1983; Beardsley et al., 1985). The ECS is also influenced by the warm and oligotrophic Taiwan Warm Currents in the south and the Kuroshio Current (from the Western Equatorial Pacific) in the east (Jiao et al., 2005). More interestingly, the ECS has also experienced serious environmental problems, including eutrophication, harmful algal blooms, and chemical pollution (Shen et al., 2011; Cheng et al., 2012; Yu et al., 2013). Because of the strong influence of its riverine inputs, currents and environmental problems, the ECS exhibits complex physical and chemical conditions and has become an ideal study area for ecological investigations of the temporal and spatial dynamics of biota (Dang et al., 2008; Xiong et al., 2014; Zhang et al., 2014). Increasing research efforts have focused on microbial ecology in the ECS, particularly the microbial community structure and diversity of organisms associated with the sulfur cycle, such as SRB and SOB (Nie et al., 2009; Wu et al., 2009; He et al., 2015); however, because of the limitations of the applied methods (DGGE or clone libraries), only a few dominant species have been identified. In the present study, the 16S rRNA gene and two functional genes (*dsrB* and *soxB*) were combined in a high-throughput sequencing analysis, and this combination allowed for an in-depth analysis of the community structure and diversity of SRB and SOB in sediment cores from the ECS. In addition, quantitative PCR (qPCR) was performed to reveal the vertical distribution of SRB and SOB populations and provide insight into the SRB and SOB community characteristics.

## Materials and Methods

### Site and Sampling Description

Two core samples from ECS sediments were collected from station S31 (length: 21 cm) and station S33 (length: 50 cm) during a research cruise in July 2011 on the R/V Run-Jiang (Supplementary Figure S1). A QuAAtro nutrient auto analyzer (Seal Analytical Ltd., United Kingdom) was used to measure the pore-water dissolved N, P, and Si concentrations of the following: nitrate (NO_3_-N), nitrite (NO_2_-N), ammonium (NH_4_-N), phosphate (PO_4_-P), and silicate (SiO_3_-Si) (Supplementary Table S1). The sampling methods as well as the methods used for the determination of environmental parameters [pH and the concentrations of Fe(II), Mn(II), sulfate and excess ^210^Pb] were described in detail by He et al. (2015) (Supplementary Table S1 and Figure S2). After collection, the samples from stations S31 and S33 were divided into three depth sections (0–4, 8–12, and 16–20 cm) and five depth sections (0–4, 8–12, 16–20, 32–36, and 46–50 cm), respectively, and subsequently frozen at -80°C for nucleic acid extraction.

### DNA Extraction

Genomic DNA was extracted from each sediment sample using the PowerSoil DNA Isolation Kit (Mo Bio Laboratories Inc., Carlsbad, CA, United States) according to the manufacturer’s recommended protocol. The extracted DNA was stored at -80°C prior to further analyses.

### High-Throughput Sequencing and Data Processing

#### Illumina HiSeq2500 (PE250) Sequencing and Analysis

The community structure, richness, and diversity of total bacteria and SRB were studied through Illumina HiSeq2500 (PE250) sequencing based on the 16S rRNA and *dsrB* genes. Fragments of the 16S rRNA gene (∼466 bp, V3–V4 region) and *dsrB* gene (∼350 bp) were amplified using the primer pairs 341F/806R and DSRp2060F/DSR4R, respectively (Supplementary Table S2); these primer pairs have been widely employed in previous studies of bacteria from various environments (Geets et al., 2006; Michelsen et al., 2014). PCR amplification was performed in a 30-μL reaction volume containing 15 μL of Phusion^®^ High-Fidelity PCR Master Mix (New England Biolabs), 0.2 μM forward and reverse primers, and approximately 10 ng of template DNA. The amplification reactions were performed in a thermal cycler (Bio-Rad T100, United States) and consisted of an initial denaturation step at 98°C for 1 min followed by 30 cycles of denaturation at 98°C for 10 s, annealing at 50°C for 30 s, elongation at 72°C for 30 s, and a final step at 72°C for 5 min. The PCR products were analyzed via 2% agarose gel electrophoresis to assess the quality and size of the resultant amplicons. The PCR products were then mixed at equidensity ratios and purified using the GeneJET Gel Extraction Kit (Thermo Fisher Scientific). 16S rRNA and *dsrB* gene libraries were subsequently prepared using the NEB Next^®^Ultra^TM^ DNA Library Prep Kit (New England Biolabs), and the libraries were sequenced on the Illumina HiSeq2500 (PE250) platform following the manufacturer’s recommendations.

Paired-end reads (16S rRNA and *dsrB*) were assigned to the samples based on their unique barcode, truncated by removing the barcode and primer sequence, and then merged using FLASH (Version 1.2.7) (Magoè and Salzberg, 2011). The merged reads were subsequently subjected to quality-based filtering with QIIME (Caporaso et al., 2010). Briefly, the reads were truncated at any site containing more than three sequential bases with a Phred quality score (Q) below 20 and any read containing ambiguous base calls and reads with less than 75% (with respect to the total read length) consecutive high-quality base calls (Wang et al., 2016) were discarded.

#### Pyrosequencing and Sequence Analysis

The *soxB* gene (∼750 bp) was amplified with the soxB693F/soxB1446B primer set, which was previously shown to yield the most successful and reliable amplification results (Supplementary Table S2) (Meyer et al., 2007). The 25-μL amplification reaction for the *soxB* gene included 4 μL of 5 × Q5 reaction buffer (New England Biolabs), 2 μL of 2.5 mM deoxynucleoside triphosphate (dNTP) mix, 5 μL of 5 × Q5 High GC Enhancer, 1 μL of each primer (10 μM), 0.25 μL of Q5 High-Fidelity DNA Polymerase (New England Biolabs), 2.5 μL of template DNA, and 8.25 μL of double-distilled H_2_O. The reactions were maintained at 98°C for 5 min for DNA denaturation; they were then subjected to 32 cycles of 98°C for 30 s, 55°C for 40 s, and 72°C for 1 min and then to a final extension at 72°C for 7 min to ensure complete amplification. The obtained PCR product was cut from a 1.5% agarose gel and purified using the AxyPrep DNA Gel Extraction Kit (Axygen, AP-GX-250). Pyrosequencing of the *soxB* gene was subsequently conducted using the 454 FLX+ platform according to the manufacturer’s recommended protocol.

The raw data were processed using the QIIME pipeline (Caporaso et al., 2010). For quality filtering, low-quality reads shorter than 150 bp, reads with an average quality score below 19, sequences with ambiguous base calls and sequences with homopolymer runs exceeding 6 bp were removed (El-Chakhtoura et al., 2015). Moreover, the barcode and primer regions were trimmed from the sequences.

#### Operational Taxonomic Unit Clusters and Taxonomic Assignment

After quality control, UPARSE (Edgar, 2013) was employed to cluster all of the clean reads into operational taxonomic units (OTUs) using 97 and 90% similarity cutoffs (Petri et al., 2001; Müller et al., 2014) for the 16S rRNA gene and functional genes (*dsrB* and *soxB*), respectively. In addition, the most abundant sequence from each OTU was selected as the representative sequence. Taxonomic information for each representative 16S rRNA and functional gene sequence was obtained by searching the Greengenes and the NCBI (National Center for Biotechnology Information) databases, respectively. Reads that did not match any sequences in the database were clustered into the unclassified group.

### Quantification of Gene Copies in Sediments

The total bacterial 16S rRNA gene, the SRB *dsrB* gene, and the SOB *soxB* gene in the sediment samples were amplified using the primers 341F/518R, DSRp2060F/DSR4R, and soxB693F/soxB1164BK145, respectively (Supplementary Table S2); these primers were demonstrated to work effectively in previous studies (Geets et al., 2006; Dang et al., 2010; Krishnani et al., 2010). The method used for quantification of the *dsrB* and 16S rRNA genes in the investigated samples has been described previously (He et al., 2015). All qPCR assays targeting the *soxB* gene were performed in triplicate using an ABI PRISM^®^ 7500 Sequence Detection System (Applied Biosystems, Foster City, CA, United States). Each 20-μL qPCR mixture contained the following reagents: 10 μL of FastStart Universal SYBR Green Master Mix (Rox) (Roche Diagnostics, Mannheim, Germany), 0.2 μg μL^-1^ of bovine serum albumin, 0.4 μM of each primer, and 2.0 μL of template DNA. The qPCR amplification conditions were as follows: 95°C for 10 min followed by 40 cycles of 15 s at 95°C, 45 s at 55°C, and 1 min at 72°C. To evaluate the specificity of the qPCR amplification, the PCR products were sequenced by the Beijing Genomics Institute. In addition, the specificity of the amplification products was verified by melting curve analysis and visualized in agarose gels. Melting curves were obtained at 60–95°C; a read was obtained after every 1°C increase in temperature, and the temperature was maintained for 1 s between reads. The resultant qPCR data were analyzed using ABI PRISM 7500 SDS software.

Plasmids containing the target gene fragments were extracted from *Escherichia coli* hosts using a FastPlasmid Mini Kit (CWBIO, Beijing, China) and quantified using a Picodrop microliter spectrophotometer (Picodrop, Saffron Walden, Essex, United Kingdom). Standard curves for the qPCR assays were obtained with serial 10-fold dilutions of reference plasmids. Standard curves with efficiencies ranging from 90 to 110% and a corresponding *R*-value greater than 0.99 were considered credible.

### Statistical Analyses

After randomly reducing the number of reads to the lowest number of reads in any individual sample, the richness indices (Chao 1 estimates), diversity indices (Shannon index), and Good’s coverage were obtained with QIIME (Caporaso et al., 2010). Furthermore, unweighted pair group method with arithmetic mean (UPGMA) clustering based on a weighted UniFrac distance matrix was conducted using QIIME to examine the differences in the bacterial communities between the sediment samples. The relationships between community composition and sediment characteristics were examined using BIOENV, detrended correspondence analysis (DCA), and redundancy analysis (RDA) with the R package vegan (Oksanen et al., 2011). The DCA analysis indicated that a linear-model-based RDA analysis was more suitable than unimodal CCA for our data (Hernández-Landa et al., 2015). BIOENV provides the subset of environmental variables that best explain the community variation among the sampling stations using Spearman rank correlation, and RDA determines the percentage of the community composition variation explained by this subset of environmental variables. The significance of the environmental variables was tested by a Monte Carlo permutation test (999 unrestricted permutations, *p* < 0.05). A Pearson’s correlation analysis between microbial abundance and environmental parameters was performed using SPSS statistics software.

### Nucleotide Sequence Accession Numbers

The sequence data generated in this study were deposited in the NCBI Short Read Archive database under accession numbers SRP077048 (16S rRNA), SRP077077 (*dsrB*), and SRP077086 (*soxB*).

## Results

### Bacterial 16S rRNA Gene Analysis

A total of 371,534 high-quality 16S rRNA gene sequences with an average length of 418 bp were obtained from an Illumina HiSeq2500 (PE250) sequencing analysis of the eight sediment samples. The high-quality sequences were clustered into OTUs with 97% sequence similarity. Accordingly, bacterial richness (Chao 1 estimator) and diversity (Shannon index) estimates were calculated for each sample and are shown in **Table 1**. Good’s coverage estimate for each sample ranged from 98.2 to 99.2%, indicating that the sampling was sufficient to cover almost all bacterial communities. A total of 1,607–2,196 OTUs with 97% similarity were identified in the eight sediment samples. The samples from S33 exhibited more OTUs than those from S31, and the surface sediment (0–4 cm) samples presented the lowest number of OTUs at each station. The diversity indices (Shannon indices) and species richness indices (Chao 1) indicated that the samples from S33 displayed greater richness and diversity than those from S31. These results revealed higher microbial diversity at S33. Furthermore, the diversity indices did not change markedly with increasing depth. In addition, the lowest and the highest richness indices were obtained for the 0–4 and 32–36 cm sections, respectively.

**Table 1:** Vertical distribution of sediment bacterial community diversity and richness estimators based on the 16S rRNA gene.

Station	Depth (cm)	Read number	OTUs	Shannon	Chao 1	Good’s coverage (%)
S31	0–4	41,663	1,607	8.091	1770.117	98.9
	8–12	50,347	1,733	7.883	1975.441	98.5
	16–20	41,059	1,708	8.048	1951.907	98.7
S33	0–4	40,919	1,766	8.496	1818.074	99.2
	8–12	46,306	1,989	8.536	2237.209	98.6
	16–20	49,152	2,123	8.573	2325.423	98.5
	32–36	51,004	2,196	8.545	2640.667	98.2
	46–50	51,084	1,975	8.589	2297.087	98.6

**FIGURE 1:**
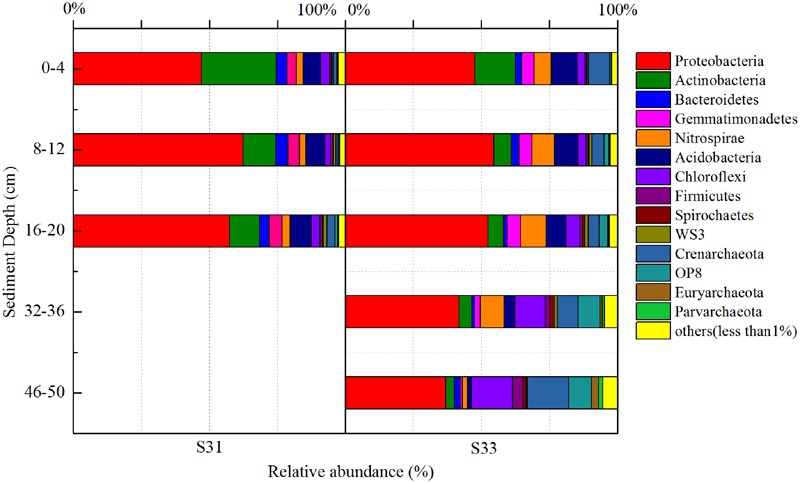
16S rRNA gene-based relative abundance of dominant bacterial groups (relative abundance >1%) observed in core samples collected from the East China Sea.

**FIGURE 2:**
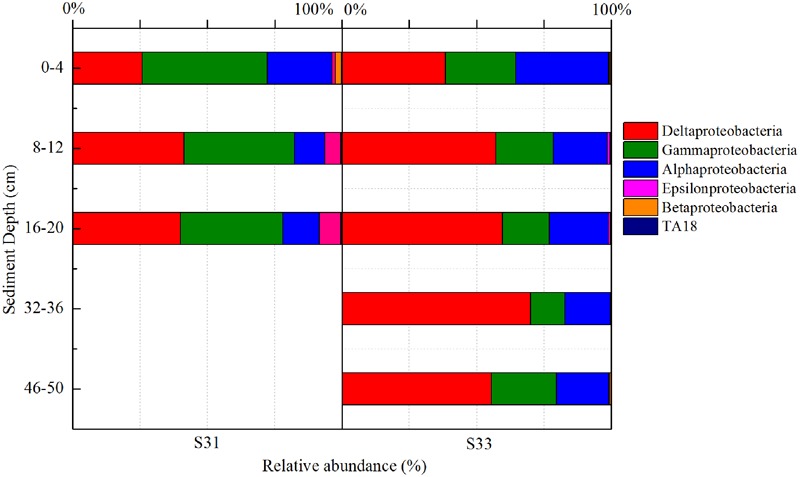
16S rRNA gene-based relative abundance of different classes of Proteobacteria in core samples collected from the East China Sea.

In total, 52 different bacterial phyla were detected across all sediment samples, and the dominant phyla (exhibiting a relative abundance >1% in at least one sample), which accounted for more than 94.57% of the total sequences in each sample, are shown in **Figure 1**. Proteobacteria was the most abundant phylum in all samples and accounted for 36.9–62.4% of all sequences. After the relative abundance of Proteobacteria reached its peak value at a depth of 8–12 cm, it exhibited a tendency to significantly decline with increasing depth at each station. Within Proteobacteria, the majority of sequences were assigned to Deltaproteobacteria (25.8–70.0%), Gammaproteobacteria (12.7–46.5%), and Alphaproteobacteria (11.1–34.3%) (**Figure 2**). Furthermore, Deltaproteobacteria and Alphaproteobacteria were more abundant at S33, whereas Gammaproteobacteria was more abundant at S31.

In the shallow sediment layers (0–20 cm) from each station, Actinobacteria and Acidobacteria were the second and third most dominant communities, accounting for 5.8–27.6 and 6.5–9.4% of all bacteria amplicons, respectively. Furthermore, the relative abundance of Actinobacteria decreased with increasing depth at each station, whereas the relative abundance of Acidobacteria increased with increasing depth at S31 and decreased with increasing depth at S33. In the deeper sediment layers (32–36 and 46–50 cm) from S33, Chloroflexi and Crenarchaeota were the second and third most dominant communities, accounting for 13.1 and 11.4% of bacterial amplicons, respectively.

**FIGURE 3:**
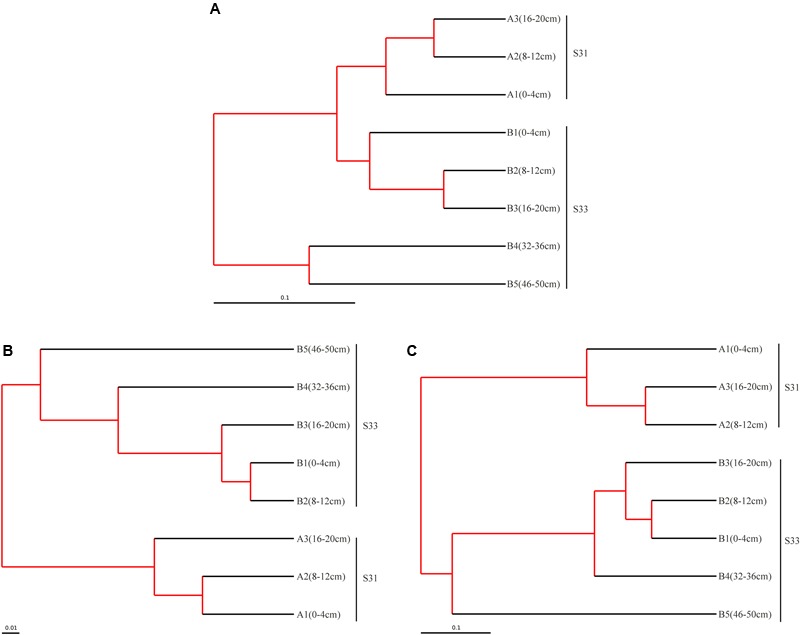
Tree representing the results of the UPGMA hierarchical clustering of the weighted UniFrac distance matrix for the 16S rRNA **(A)**, *dsrB* **(B)**, and *soxB* **(C)** genes in sediments from the ECS. The scale bar indicates the distance between clusters in UniFrac units. The red branch represents 75–100% level of jackknife support.

Above all, the bacterial communities at the two investigated stations showed similarities in their dominant groups of phyla, particularly in shallow sediment layers. However, certain variations, such as differences in class groups, were observed between the two stations. The similarities and dissimilarities between the bacterial communities in the different sediment samples were further quantified through UPGMA clustering analysis based on the weighted UniFrac distance metric (**Figure 3A**). Overall, the bacterial community structures in the shallow sediment layers (0–20 cm) from S31 and S33 were similar, and those in the deeper sediment layers (32–36 and 46–50 cm) from S33 clustered separately. These findings, which showed high jackknife support, suggest that sampling depth plays a relatively important role in bacterial community structure. However, analysis of the cluster containing the shallow sediment samples revealed that the samples from S31 formed a separate group and clustered away from the samples from S33, revealing the existence of differences in bacterial community structure between the two sampling sites.

### Functional Gene Analysis

#### Functional Gene Diversity

The total numbers of high-quality sequences from the *dsrB* and *soxB* genes obtained from all samples were 615,062 and 107,688, respectively. Using a similarity cutoff of 90%, these sequences were classified into 829 OTUs for the *dsrB* gene and 380 OTUs for the *soxB* gene. The corresponding species richness (Chao 1 estimator) and diversity estimates (Shannon index) were calculated for each sample and are listed in **Tables 2A,B**. Good’s coverage values of the functional genes were all above 99%, indicating that the obtained sequences could adequately reflect the diversity of SRB and SOB in the sediments. Overall, the *soxB* gene diversity was substantially higher than that of the *dsrB* gene, whereas the *dsrB* gene richness was markedly higher than that of the *soxB* gene.

**Table 2A:** Similarity-based OTUs and species richness and diversity estimates based on the *dsrB* gene.

Station	Depth (cm)	Read number	OTUs	Shannon	Chao 1	Good’s coverage (%)
S31	0–4	79,321	336	3.745	367.089	99.9
	8–12	78,861	240	3.782	266.464	99.9
	16–20	76,887	322	3.755	360.936	99.9
S33	0–4	67,284	336	2.708	343.483	99.9
	8–12	78,034	393	2.885	425.697	99.9
	16–20	76,646	456	3.247	485.94	99.9
	32–36	82,929	487	4.240	509.685	99.9
	46–50	75,100	413	4.27	426.448	99.9

**Table 2B:** Similarity-based OTUs and species richness and diversity estimates based on the *soxB* gene.

Station	Depth (cm)	Read number	OTUs	Shannon	Chao 1	Good’s coverage (%)
S31	0–4	12,100	180	5.060	185.571	99.8
	8–12	9,202	128	4.934	145.400	99.8
	16–20	9,070	103	4.057	115.720	99.8
S33	0–4	12,694	148	5.295	152.119	99.8
	8–12	12,817	141	4.845	142.747	99.9
	16–20	11,052	145	4.914	151.037	99.8
	32–36	18,786	113	5.086	110.563	99.9
	46–50	21,967	182	5.985	193.256	99.7

The diversity indices of the *dsrB* gene did not change markedly with increasing depth at S31 and increased with increasing depth at S33. Moreover, the diversity indices of the *dsrB* gene in the shallow sediment layers (0–20 cm) from S31 were greater than those from S33 and lower than those obtained for the deeper sediment layers (32–36 and 40–46 cm) from S33. The richness of the *dsrB* gene was lowest in the 8–12 cm sediment depth range at station S31 and reached its peak value in the depth range of 32–36 cm at station S33. For the *soxB* gene, the diversity and richness indices decreased with increasing depth at S31, whereas at S33, the diversity and richness indices decreased with increasing depth until suddenly increasing and peaking in the depth range of 46–50 cm.

#### Functional Gene Community Composition

##### *dsrB* gene

**FIGURE 4:**
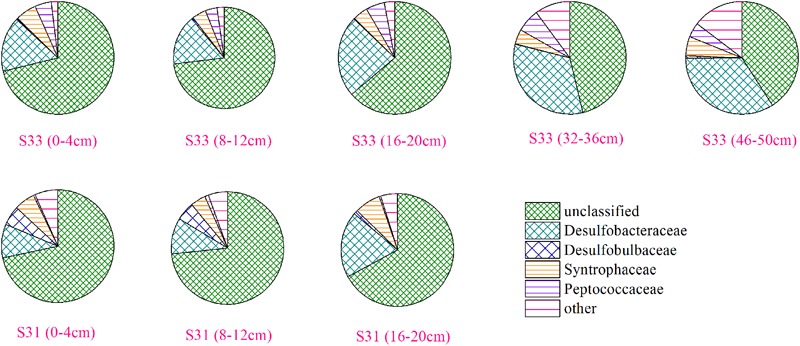
Community structure of the *dsrB* gene in sediment samples at the family level.

The community structure of the *dsrB* gene in the samples was analyzed based on the 109 dominant (showing >1% abundance in at least one sample) and core (common to all samples) OTUs (representing 85.3–98.2% of the total sequences). Overall, 103 OTUs, accounting for 81.1–94.7% of the total *dsrB* sequences, were affiliated with Deltaproteobacteria, and six OTUs, representing 0.45–6.6% of the total *dsrB* sequences, were classified as belonging to Firmicutes. As shown in **Figure 4**, among the dominant Deltaproteobacteria, the majority of sequences (15 OTUs with relative abundances ranging from 40.91 to 73.36%) could not be assigned at the class or family levels. The 42 OTUs belonging to the Desulfobacteraceae family (ranging from 9.60 to 34.05%) represented a significant fraction and tended to increase with increasing depth at each station. A total of 42 OTUs with relative abundances ranging from 4.23 to 7.49% were affiliated with Syntrophaceae. The Desulfobulbaceae family (four OTUs) reached an abundance of 4.06% on average at S31 but only accounted for 0.40% of the sequences at S33.

**FIGURE 5:**
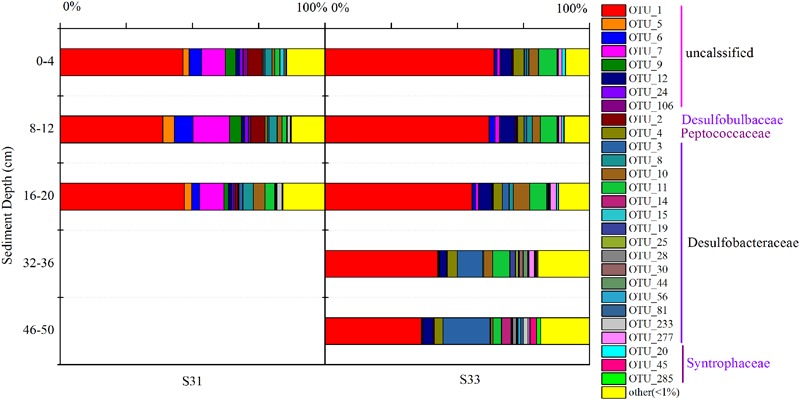
Relative abundance of dominant OTUs (relative abundance >1%) of the *dsrB* gene observed in core samples collected from the East China Sea.

The relative abundance of all dominant OTUs (total of 28 OTUs) from each sample exceeded 80%, indicating that the community composition of the *dsrB* gene in sediments was well reflected by the dominant OTUs. Thus, the distribution of dominant OTUs was analyzed to better understand the composition and structure of the *dsrB* gene in the samples (**Figure 5**). OTU1, showing relative abundances ranging from 38.8 to 63.8%, was the predominant SRB group in all sediment layers. Moreover, the relative abundance of OTU1 decreased with increasing depth at S33, whereas at S31, no significant difference was observed between the depth ranges of 0–4, 16–20, and 8–12 cm. In addition, the relative abundance of OTU1 in the shallow sediment layers (0–20 cm) from S33 was greater than that in the samples from S31. OTU2 reached an average abundance of 3.98% at S31 but only accounted for 0.07–0.5% of the sequences at S33. A similar trend of differences was observed for OTU5, OTU6, OTU7, OTU8, and OTU9, whereas OTU3, OTU4, OTU10, OTU11, and OTU12 were more abundant at S33 than at S31.

Taken together, the results regarding the distribution of dominant OTUs at S31 and S33 indicated certain differences. Interestingly, similar results were obtained via UPGMA clustering analysis (**Figure 3B**). The UPGMA analysis showed that the SRB communities at S31 and S33 clustered separately from each other, revealing the dissimilarity in the SRB community structure between the two sampling stations and among different sediment depths.

##### *soxB* gene

**FIGURE 6:**
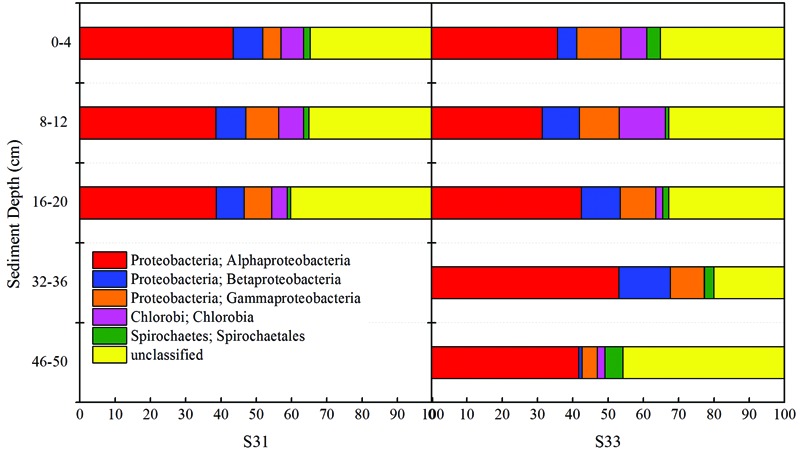
Relative abundance of the *soxB* gene observed in core samples collected from the East China Sea.

Taxonomic classification revealed that Proteobacteria accounted for the most abundant clades at the phylum level, representing 46.91–77.17% (131 OTUs) of the sequences. Chlorobi and Spirochaetes were the second and third most dominant phyla, accounting for 5.32 (two OTUs) and 2.31% (eight OTUs) of the sequences, respectively (**Figure 6**). In addition, 238 OTUs representing 20.05–45.82% of the sequences were unclassified (**Figure 6**). The dominant Proteobacteria phylum was classified into three classes: Alphaproteobacteria (varying from 31.37 to 53.00%), Betaproteobacteria (varying from 1.00 to 14.64%), and Gammaproteobacteria (varying from 4.28 to 12.50%).

**FIGURE 7:**
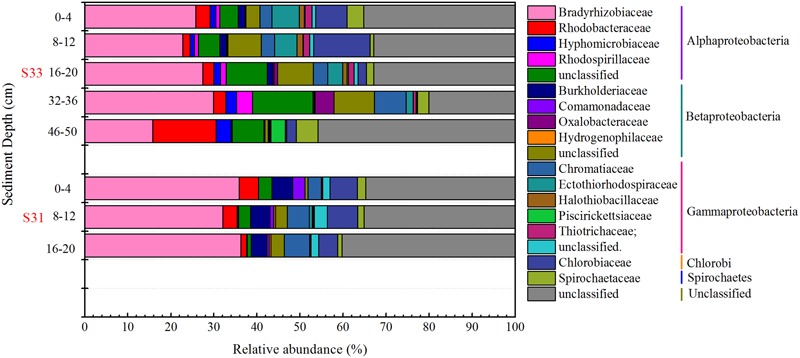
Relative abundance of the *soxB* gene in the core samples at the family level.

The composition and structure of the SOB community in the various samples were compared at the family level (**Figure 7**). Bradyrhizobiaceae was the dominant family in all samples and was more abundant (34.72%) in S31 than in S33 (ranging from 15.79 to 29.86%). Four other minor families (Rhodobacteraceae, Hyphomicrobiaceae, Rhodospirillaceae, and an unclassified group) within Alphaproteobacteria were more abundant in S33 than in S31. Among the Betaproteobacteria, Burkholderiaceae, with a relative abundance of 4.31% (on average), was the dominant family in S31, whereas an unclassified group with a relative abundance of 5.91% (on average) was the dominant family in S33. Furthermore, within Gammaproteobacteria, Ectothiorhodospiraceae was the dominant family in almost all samples from S33, but its abundance was only 0.11–0.71% at S31. The UPGMA clustering analysis (**Figure 3C**) based on the weighted UniFrac distance metric showed that the SOB communities from S31 and S33 clustered separately from each other, further demonstrating the differences in the SOB community structure between the two sampling stations and among different sediment depths.

### Influence of Environmental Variables on Total Bacterial, SRB, and SOB Communities

**FIGURE 8:**
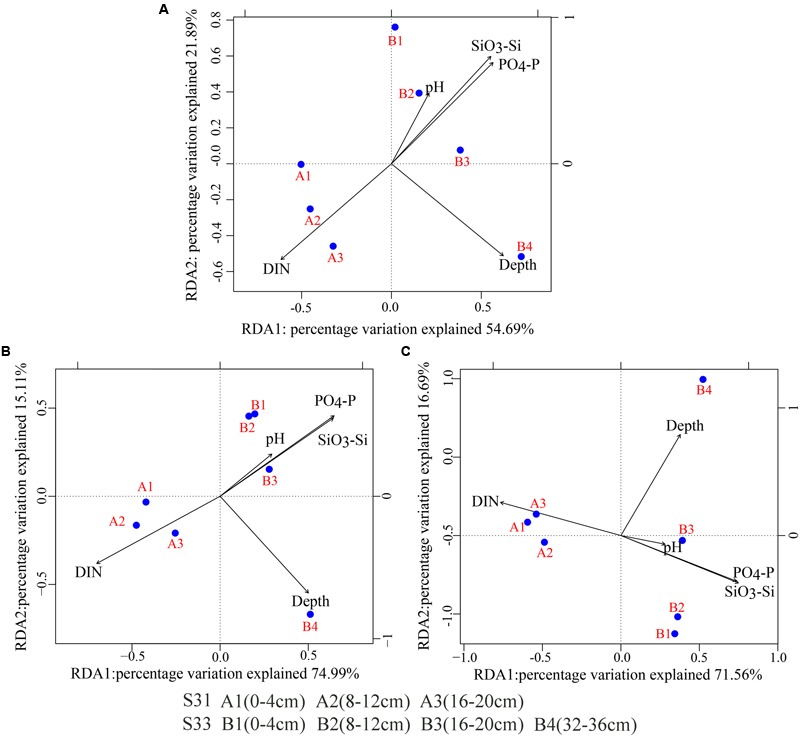
Redundancy analysis (RDA) biplot of environmental parameters and the 16S rRNA and functional genes (*dsrB* and *soxB*). **(A)** 16S rRNA; **(B)** *dsrB*; **(C)** *soxB*.

BIOENV identified that the environmental factors most strongly correlated with the total bacteria, SRB, and SOB communities were depth, pH, dissolved inorganic nitrogen (DIN), phosphate (PO_4_-P), and silicate (SiO_3_-Si) (correlation >0.6). Together, these variables explained 76.58, 90.10, and 88.25% of the variation in the bacteria, SRB, and SOB communities, respectively, as determined through RDA (**Figure 8**). The environmental variables that were found to contribute significantly to the microbial community–environment relationship (900 Monte Carlo permutations) were depth and DIN for all of the examined genes (16S rRNA, *dsrB* and *soxB* gene).

### Abundance of Total Bacterial 16S rRNA and Functional Genes

**FIGURE 9:**
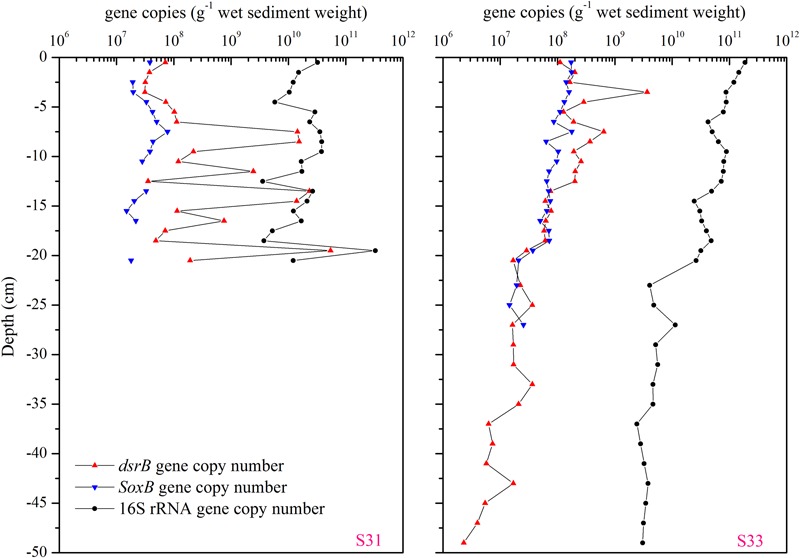
Vertical profiles of the *dsrB, soxB*, and total bacterial 16S rRNA gene copy numbers in the core samples from S31 and S33.

The abundance of total bacterial 16S rRNA and functional genes (*dsrB* and *soxB*) in the 21- and 50-cm-long core samples from S31 and S33, respectively, were determined via qPCR (**Figure 9**). In the core sample from S31, the vertical abundance profile of the *dsrB, soxB*, and 16S rRNA genes showed marked fluctuations, and the range of fluctuation in the abundances of the 16S rRNA and *soxB* genes was smaller than that in the abundance of the *dsrB* gene. However, analysis of the core sample from S33 showed that the abundances of the *dsrB, soxB*, and 16S rRNA genes gradually decreased with increasing depth.

## Discussion

In this study, a comparative analysis of the sulfur cycle-related microbial communities (SRB and SOB) in sediment cores was performed using an approach combining 16S rRNA, *dsrB*, and *soxB* gene high-throughput sequencing with qPCR analyses. The combination of these molecular techniques provided a detailed description of the SRB and SOB communities. The traditional approaches (e.g., DGGE and clone library), which have been widely applied for the detection of SRB and SOB in various environments, might notably underestimate their diversity. High-throughput sequencing based on functional genes (*dsrB* and *soxB*) can result in a greater number of sequences and almost complete coverage (Good’s coverage values were all greater than 99%), providing more comprehensive information regarding the diversity and community composition of the SRB and SOB communities. Thus, the diversity, richness and OTU numbers observed in our high-throughput sequencing analyses were notably greater than those obtained in previous studies that employed other molecular methods (Jiang et al., 2009; Yang et al., 2013; Yousuf et al., 2014; He et al., 2015). Further analysis indicated that rare species make important contributions to the greater richness and diversity of the SRB and SOB communities. For instance, as shown in **Table 3A**, the number of dominant OTUs in each sample was similar, ranging from 8 to 13 and representing 3.08% of OTUs (on average); however, these OTUs comprised more than 77% of the sequence abundance for the *dsrB* gene. Although the rare OTUs (relative abundance <0.01%) (Galand et al., 2009) accounted for 54.03% of OTUs (on average), they only comprised 0.84% (on average) of the sequence abundance. Similar results were obtained for the *soxB* gene (**Table 3B**).

**Table 3A:** Rare and dominant OTUs based on the *dsrB* gene.

Station	Depth (cm)	Total number of OTUs	Dominant OTUs	Abundance of dominant OTUs (%)	Rare OTUs	Abundance of rare OTUs (%)
S31	0–4	336	13	83.54	183	0.78
	8–12	240	12	84.23	121	0.49
	16–20	322	12	81.41	180	0.70
S33	0–4	336	8	86.84	191	0.77
	8–12	393	9	86.70	257	1.03
	16–20	456	10	85.30	287	1.23
	32–36	487	11	77.16	220	0.97
	46–50	413	12	77.48	180	0.78

**Table 3B:** Rare and dominant OTUs based on the *soxB* gene.

Station	Depth (cm)	Total number of OTUs	Dominant OTUs	Abundance of dominant OTUs (%)	Rare OTUs	Abundance of rare OTUs (%)
S31	0–4	180	18	76.62	88	4.13
	8–12	128	19	78.45	47	1.76
	16–20	103	14	85.06	49	2.05
S33	0–4	148	23	76.96	61	2.53
	8–12	141	20	80.09	59	2.69
	16–20	145	21	76.78	59	2.51
	32–36	113	22	80.58	26	1.22
	46–50	182	30	67.58	50	1.72

The 16S rRNA sequencing analysis identified 235 and 191 OTUs as belonging to potential SRB and SOB, respectively, based on family species classification. Furthermore, these SRB OTUs were affiliated with 10 families, accounting for 7.63–19.62% (14.12% on average) of the total 16S rRNA gene sequences (**Table 4A**), and the SOB OTUs were affiliated with 12 families, accounting for 5.99–18.78% (12.53% on average) of the total 16S rRNA sequences (**Table 4B**). This finding is consistent with the results of a previous study in this region that found that numerous sequences were affiliated with potential SRB and SOB (Ye et al., 2016). Overall, these results confirm the high abundance of potential SOB and SRB groups in the ECS sediments and suggest the existence of an active sulfur cycle in this area, and these sulfur cycle-related bacterial communities have considerable potential for further exploration.

**Table 4A:** SRB community composition based on taxonomic information of the *dsrB* and 16S rRNA genes.

Family level	OTUs		Total abundance (%)	
	16S rRNA	*dsrB*	16S rRNA	*dsrB*
Peptococcaceae	6	6	0–0.33	0.45–6.60
Desulfobacteraceae	51	42	1.26–4.97	9.60–34.05
Desulfobulbaceae	38	4	0.27–9.37	0.11–5.67
Syntrophaceae	6	42	0.007–0.34	4.23–7.50
Nitrosomonadaceae	1	–	0.01–0.62	–
Desulfarculaceae	26	–	0.32–5.82	–
Desulfovibrionaceae	1	–	0–0.01	–
Syntrophobacteraceae	52	–	0.13–0.77	–
Nitrospiraceae	12	–	0.10–1.16	–
Thermodesulfovibrionaceae	42	–	1.72–8.59	–

*–, Not detected.*

**Table 4B:** SOB community composition based on taxonomic information of the *soxB* and 16S rRNA genes.

Family level	OTUs		Total abundance (%)	
	16S rRNA	*soxB*	16S rRNA	*soxB*
Spirochaetaceae	30	8	0.03–0.69	0.85–5.04
Chlorobiaceae	–	2	–	0.13–13.08
Helicobacteraceae	5	–	0.02–4.57	–
Rhodospirillaceae	42	5	0.24–1.43	0.12–3.75
Rhodobacteraceae	27	20	0.27–3.32	1.33–14.74
Bradyrhizobiaceae	1	35	0–0.01	15.79–36.26
Hyphomicrobiaceae	16	2	1.38–2.67	0.01–3.51
Rhizobiaceae	1	–	0–0.01	–
Neisseriaceae	1	–	0–0.03	–
Comamonadaceae	7	3	0.02–0.15	0.05–2.88
Ectothiorhodospiraceae	10	2	0.07–0.56	0.09–6.38
Thiotrichaceae	7	2	0–0.07	0.06–1.60
Piscirickettsiaceae	44	4	0.52–13.67	0–3.38
Burkholderiaceae	–	6	–	0.13–4.82
Oxalobacteraceae	–	1	–	0–4.36
Hydrogenophilaceae	–	1	–	0–0.44
Chromatiaceae	–	18	–	0.25–7.39
Halothiobacillaceae	–	2	–	0–1.42

*–, Not detected.*

The *dsrB* gene sequencing analysis performed in this study identified two phyla (Proteobacteria and Firmicutes), and Deltaproteobacteria (within Proteobacteria) represented the dominant SRB. Our data were consistent with previous observations obtained from various environments, such as marsh sediments (Jemaneh et al., 2013), the Great Salt Lake (Kjeldsen et al., 2007), mangrove sediments (Varon-Lopez et al., 2013), wastewater treatment plants (Biswas et al., 2014), and marine and estuary sediments (Jiang et al., 2009; Wu et al., 2009; He et al., 2015), suggesting that Deltaproteobacteria have a wide adaptation range and play major roles in global sulfur cycling. The *soxB* gene sequencing results revealed that the SOB community was dominated by Alphaproteobacteria and unclassified groups, followed by Gammaproteobacteria and Betaproteobacteria. For comparison, Gammaproteobacteria and Epsilonproteobacteria are considered dominant Sox organisms in marine sediments (Sievert et al., 2008; Lenk et al., 2011; Akerman et al., 2013; Dyksma et al., 2016); however, no Epsilonproteobacteria groups were observed in the present study. This discrepancy could be due to primer bias; the primers used for *soxB* gene amplification amplify the *soxB* genes of Epsilonproteobacteria and Chloroflexi very poorly (Meyer and Kuever, 2007). In addition, Bradyrhizobiaceae within Alphaproteobacteria and Burkholderiaceae within Betaproteobacteria were the dominant families in our study area; however, previous research revealed that these families might adapt to oligotrophic sulfur environments, such as soil (Jung et al., 2005; Masuda et al., 2010) and rhizosphere soils (Anandham et al., 2008; Masuda et al., 2016). Therefore, further study is required to determine whether the families described here are the result of contamination or actually exist. More interestingly, most of the domain and core OTUs obtained through *dsrB* and *soxB* gene high-throughput sequencing were difficult to clearly assign at the family or genus level, partially due to the limited number of reference sequences. For instance, 15 OTUs for the *dsrB* gene, with a relative abundance ranging from 40.91 to 73.36%, and 238 OTUs for the *soxB* gene, with a relative abundance ranging from 20.05 to 45.82%, could not be assigned at the family or genus level. Overall, these findings suggest that the ECS environment contains a high abundance of yet unknown SRB and SOB lineages, which should be studied in more detail. Indeed, this phenomenon is consistent with the following recent findings: 62% of *dsrB* sequences in surface sediments from the ECS exhibit low similarity with previously cultured SRB (Zhang et al., 2016); 57.6% of *dsrAB* clones from polluted harbor sediments belong to unclassified groups of SRB (Zhang et al., 2008); and 60% of sequences obtained through a *dsrA* gene analysis show no clear affiliation with a known SRB (Quillet et al., 2012). Furthermore, similar results have been reported by Yousuf et al. (2014) for *soxB*. In addition, we noticed that these unclassified OTUs exhibit high similarity with environmental *dsrB*/*soxB* sequences, as demonstrated through BLAST identity searches. For example, most of the unclassified *dsrB* OTUs displayed high similarities (>90%) with uncultured SRB recovered from Japanese fish farm sediments (Kondo et al., 2012) and ECS sediments (He et al., 2015; Zhang et al., 2016). Similarly, most of the unclassified *soxB* sequences exhibited high similarity (>80%) with sequences recovered from coastal soil ecosystems (Yousuf et al., 2014). These observations suggest that these unclassified sequences are widely distributed in marine and terrestrial environments and play a vital role in the biogeochemical cycling of sulfur and carbon.

A comparison of the SRB community composition identified based on the 16S rRNA and *dsrB* genes showed that 16S rRNA gene sequencing detected 10 SRB families, whereas *dsrB* gene sequencing only detected four SRB families (**Tables 4A,B**). Moreover, it was difficult to obtain more detailed taxonomic information for the SRB community based on the 16S rRNA and *dsrB* genes. Overall, the *dsrB* gene proved to be less efficient for the proper annotation of taxonomic information for SRB groups. This phenomenon might be attributed to the limited number of reference sequences used to annotate taxonomic information for the *dsrB* gene. In contrast, the results obtained from the high-throughput sequencing of the *soxB* gene revealed 15 SOB families, whereas the high-throughput sequencing of the 16S rRNA gene revealed 12 SOB families. In addition, the *soxB* gene can provide more detailed taxonomic information (at the genus or even the species level) for the SOB community (data not shown) than the 16S rRNA gene. Taken together, the results indicate that the *soxB* gene might better define SOB groups.

The 16S rRNA, *dsrB* and *soxB* gene high-throughput sequencing data obtained in the present study revealed a high diversity of SRB and SOB in sediments from the ECS, and the classification of SRB and SOB communities based on 16S rRNA gene and functional gene was analyzed and compared according to the results of a previous study (Meyer et al., 2016). Overall, the present study provided more comprehensive information regarding the SRB and SOB community characteristics and suggested the existence of an active sulfur cycle in the study area. Significantly, the sulfate amount never decreases below approximately 24 mM in the core samples, which appears to contradict the conclusion that SRB and SOB communities are responsible for active sulfur cycling. In fact, this phenomenon will be observed based on the fact that the sulfate consumed by sulfate reduction is recycled within sediments by oxidation of reduced inorganic sulfur compounds to sulfate. As previously reported, 80–95% of the massive amount of hydrogen sulfide formed through sulfate reduction is recycled within sediments and gradually oxidized back to sulfate (Jørgensen et al., 1990; Jørgensen and Nelson, 2004). Jørgensen et al. (1990) reported that sulfate reduction rates increased in the top 10 cm of core in the Belt Sea; however, the concentration of sulfate varied little (approximately 25 mM). Similarly, Leloup et al. (2009) revealed that substantial sulfate reduction rates were measured in the top 25 cm of core, and the sulfate concentration profile was approximately 20 mM. However, the diversity of sulfur cycle-related bacteria and the potential sulfur cycle activity might still be overestimated or underestimated because certain named SRB/SOB might did not actually take part in the sulfur cycle in sediments, such as *Pelotomaculum* and *Sporotomaculum*, which are not able to grow with sulfite and/or sulfate as electron acceptors even though they are closely affiliated with SRB (Brauman et al., 1998; Imachi et al., 2006). Instead, certain unnamed SRB/SOB, such as archaeal anaerobic methanotrophs, might be involved in the sulfur cycle in sediments (Treude et al., 2014). Fortunately, these groups are only found in special habitats and account for a small portion of the identified communities (Brauman et al., 1998; Imachi et al., 2006; Miyashita et al., 2009; Treude et al., 2014). Thus, the use of 16S rRNA and functional genes (*dsrB* and *soxB*) as proxies for determining the microbial groups that play active roles in sulfur cycling and for studying the diversity of SOB and SRB is a powerful approach.

The sediment environments of the ECS are highly heterogeneous (Dang et al., 2008; Fang et al., 2013); this characteristic has a strong influence on the spatial heterogeneity of the sediment microbial communities in the ECS, as observed in previous studies (Dang et al., 2008; Feng et al., 2009; Chen et al., 2014; Ye et al., 2016). Based on the sediment grain size combined with the overlying water masses, the ECS can be divided into three domains: the inner shelf mud area, the outer shelf sand area, and the slope plus Okinawa Trough mud area (Liu et al., 2006; Xu et al., 2016). S31 is located within the inner shelf mud deposits along the Zhejiang coast, whereas S33 is located at the periphery of the Zhejiang coastal mud area (Supplementary Figure S1). Thus, most of the environmental parameters showed substantial variation among the different samples and among the various sediment depths (Supplementary Table S1 and Figure S2). The concentration of DIN, for instance, ranged from 91.44 to 449.21 μM in station S31 and from 18.81 to 120.73 μM in station S33 (Supplementary Table S2). ^210^Pb has been proven to serve as an effective proxy for sedimentary dynamics processes, such as erosion–transportation–deposition, boundary scavenging, and resuspension process, in coastal and shelf regions (Huh and Su, 1999; Su and Huh, 2002). The excess ^210^Pb activity varied markedly between the two sediments (Supplementary Figure S2). At station S31, ^210^Pb_ex_ activity was stable from the surface sediment to a depth of 8 cm; however, at a depth below 12 cm, ^210^Pb_ex_ activity increased gradually and displayed characteristics of reverse accumulation, indicating that this sedimentary layer has experienced sedimentary dynamic events. At station S33, ^210^Pb_ex_ activity remained stable until a depth of 5 cm and then gradually declined. This finding indicates that the sedimentary dynamic environment at S33 was more stable than that at S31, as observed in our previous study (He et al., 2015). Our results showed that the abundances of 16S rRNA, *dsrB* and *soxB* genes show substantial fluctuations at S31 (**Figure 9**), and none of the gene abundance values were significantly correlated with sediment depth. In contrast, all of the genes (16S rRNA, *dsrB* and *soxB*) gradually decreased with increasing depth at S33 (**Figure 9**) and were significantly negatively correlated with sediment depth. These results suggest that the sedimentary dynamic environment is likely an important factor in controlling the vertical distribution of the abundances of total bacterial 16S rRNA gene and functional genes (*dsrB* and *soxB*). Further analysis revealed similar vertical fluctuations in the abundance of the 16S rRNA, *dsrB* and *soxB* genes at S31, but the range of fluctuation obtained for the abundance of the 16S rRNA and *soxB* genes was smaller than that observed for the *dsrB* gene. In addition, the ratios of the functional genes (*dsrB* and *soxB*) to the total bacterial 16S rRNA gene were calculated: for the *dsrB* gene, the ratio ranged from 0.22 to 40.56% in S31 and from 0.06 to 4.2% in S33, and the ratios calculated for the *soxB* gene were less than 1% at both stations. These results indicate that the abundance of SRB might be more sensitive to the sedimentary dynamic environment than that of SOB and total bacteria.

The UPGMA clustering analysis results demonstrated different community structures of total bacteria, SRB, and SOB at the two sampling sites and among the different sediment depths, which might be due to the differences in the sediment depths and sedimentary dynamic environments between the two sampling stations. Overall, the sedimentary dynamic environment is most likely related to unmeasured variables, such as salinity, organic matter, dissolved oxygen, temperature, and H_2_S, which are widely accepted as important factors in controlling the community structures of total bacteria and sulfur-cycling bacteria (SRB and SOB) (Headd and Engel, 2013; Ye et al., 2016; Zhang et al., 2016). In addition, an RDA analysis revealed that sediment depth and DIN have a significant influence on the community structure of total bacteria, SRB, and SOB. In summary, our results suggest that environmental parameters, such as sediment depth and DIN, and the sedimentary dynamic environment, which showed differences between the two sampling stations, can significantly influence the community structure of total bacteria, SRB, and SOB.

## Author Contributions

YZhe, TM, and ZY conceived and designed the experiments; YZha, XW, and HH performed the experiments and analyzed the data; YZha and YZhe wrote the paper.

## Conflict of Interest Statement

The authors declare that the research was conducted in the absence of any commercial or financial relationships that could be construed as a potential conflict of interest.

## References

[B1] Akerman N. H. Butterfield D. A. Huber J. A. (2013). Phylogenetic diversity and functional gene patterns of sulfur-oxidizing subseafloor *Epsilonproteobacteria* in diffuse hydrothermal vent fluids. *Front. Microbiol.* 4:185. 10.3389/fmicb.2013.00185 23847608 PMC3703533

[B2] Anandham R. Indiragandhi P. Madhaiyan M. Ryu K. Y. Jee H. J. Sa T. M. (2008). Chemolithoautotrophic oxidation of thiosulfate and phylogenetic distribution of sulfur oxidation gene *(sox*B) in rhizobacteria isolated from crop plants. *Res. Microbiol.* 159 579–589. 10.1016/j.resmic.2008.08.007 18832027

[B3] Aoki M. Kakiuchi R. Yamaguchi T. Takai K. Inagaki F. Imachi H. (2015). Phylogenetic diversity of *aprA* genes in subseafloor sediments on the northwestern Pacific margin off Japan. *Microbes Environ.* 30 276–280. 10.1264/jsme2.ME15023 26156553 PMC4567568

[B4] Beardsley R. C. Limeburner R. Yu H. Cannon G. A. (1985). Discharge of the Changjiang (Yangtze River) into the East China Sea. *Cont. Shelf Res.* 4 57–76. 10.1016/0278-4343(85)90022-6

[B5] Biswas K. Taylor M. W. Turner S. J. (2014). *dsrAB-*based analysis of sulphate-reducing bacteria in moving bed biofilm reactor (MBBR) wastewater treatment plants. *Appl. Microbiol. Biotechnol.* 98 7211–7222. 10.1007/s00253-014-5769-5 24788329

[B6] Bowles M. W. Mogollón J. M. Kasten S. Zabel M. Hinrichs K. (2014). Global rates of marine sulfate reduction and implications for sub-sea-floor metabolic activities. *Science* 344 889–891. 10.1126/science.1249213 24812207

[B7] Brauman A. Müller J. A. Garcia J. L. Brune A. Schink B. (1998). Fermentative degradation of 3-hydroxybenzoate in pure culture by a novel strictly anaerobic bacterium, *Sporotomaculum hydroxybenzoicum* gen. nov. sp. nov. *Int. J. Syst. Bacteriol.* 48 215–221. 10.1099/00207713-48-1-215 9542091

[B8] Caporaso J. G. Kuczynski J. Stombaugh J. Bittinger K. Bushman F. D. Costello E. K. (2010). QIIME allows analysis of high-throughput community sequencing data. *Nat. Methods* 7 335–336. 10.1038/nmeth.f.303 20383131 PMC3156573

[B9] Chen Y. Y. Zhen Y. He H. Lu X. L. Mi T. Z. Yu Z. G. (2014). Diversity, abundance, and spatial distribution of ammonia-oxidizing β-proteobacteria in sediments from Changjiang estuary and its adjacent area in East China Sea. *Microb. Ecol.* 67 788–803. 10.1007/s00248-013-0341-x 24362769

[B10] Cheng F. Song X. Yu Z. Liu D. (2012). Historical records of eutrophication in Changjiang (Yangtze) River estuary and its adjacent East China Sea. *Biogeosci. Discuss* 9 6261–6291. 10.5194/bgd-9-6261-2012

[B11] Cui J. Chen X. P. Nie M. Fang S. B. Tang B. P. Quan Z. X. (2017). Effects of *Spartina alterniflora* invasion on the abundance, diversity, and community structure of sulfate reducing bacteria along a successional gradient of coastal salt marshes in China. *Wetlands* 37 221–232. 10.1007/s13157-016-0860-6

[B12] Dang H. Y. Li J. Chen R. P. Wang L. Guo L. Z. Zhang Z. N. (2010). Diversity, abundance, and spatial distribution of sediment ammonia-oxidizing *Betaproteobacteria* in response to environmental gradients and coastal eutrophication in Jiaozhou Bay, China. *Appl. Environ. Microbiol.* 76 4691–4702. 10.1128/AEM.02563-09 20511433 PMC2901714

[B13] Dang H. Y. Zhang X. X. Sun J. Li T. G. Zhang Z. N. Yang G. P. (2008). Diversity and spatial distribution of sediment ammonia-oxidizing crenarchaeota in response to estuarine and environmental gradients in the Changjiang Estuary and East China Sea. *Microbiology* 154 2084–2095. 10.1099/mic.0.2007/013581-0 18599836

[B14] Dyksma S. Bischof K. Fuchs B. M. Hoffmann K. Meier D. Meyerdierks A. (2016). Ubiquitous *Gammaproteobacteria* dominate dark carbon fixation in coastal sediments. *ISME J.* 10 1939–1953. 10.1038/ISMEJ.2015.257 26872043 PMC4872838

[B15] Edgar R. C. (2013). UPARSE: highly accurate OTU sequences from microbial amplicon reads. *Nat. Methods* 10 996–998. 10.1038/nmeth.2604 23955772

[B16] El-Chakhtoura J. Prest E. Saikaly P. Loosdrecht M. V. Hammes F. Vrouwenvelder H. (2015). Dynamics of bacterial communities before and after distribution in a full-scale drinking water network. *Water Res.* 74 180–190. 10.1016/j.watres.2015.02.015 25732558

[B17] Fang Z. M. Yang W. F. Zhang X. X. Chen M. Fan D. J. Ma Q. (2013). Sedimentation and lateral transport of 210Pb over the East China Sea Shelf. *J. Radioanal. Nucl. Chem.* 298 739–748. 10.1007/s10967-013-2561-4

[B18] Feng B. W. Li X. R. Wang J. H. Hu Z. Y. Meng H. Xiang L. Y. (2009). Bacterial diversity of water and sediment in the Changjiang Estuary and coastal area of the East China Sea. *FEMS Microbiol. Ecol.* 70 80–92. 10.1111/j.1574-6941.2009.00772.x 19780829

[B19] Foti M. Sorokin D. Y. Lomans B. Mussman M. Zacharova E. E. Pimenov N. Y. (2007). Diversity, activity, and abundance of sulfate-reducing bacteria in saline and hypersaline soda lakes. *Appl. Environ. Microbiol.* 73 2093–2100. 10.1128/AEM.02622-06 17308191 PMC1855663

[B20] Galand P. E. Casamayor E. O. Kirchman D. L. Lovejoy C. (2009). Ecology of the rare microbial biosphere of the Arctic Ocean. *Proc. Natl. Acad. Sci. U.S.A.* 106 22427–22432. 10.1073/pnas.0908284106 20018741 PMC2796907

[B21] Geets J. Borremans B. Diels L. Springael D. Vangronsveld J. van der Lelie D. (2006). *DsrB* gene-based DGGE for community and diversity surveys of sulfate-reducing bacteria. *J. Microbiol. Methods* 66 194–205. 10.1016/j.mimet.2005.11.002 16337704

[B22] Ghosh W. Dam B. (2009). Biochemistry and molecular biology of lithotrophic sulfur oxidation by taxonomically and ecologically diverse bacteria and archaea. *FEMS Microbiol. Rev.* 33 999–1043. 10.1111/j.1574-6976.2009.00187.x 19645821

[B23] He H. Zhen Y. Mi T. Z. Xu B. C. Wang G. S. Zhang Y. (2015). Community composition and distribution of sulfate-and sulfite-reducing prokaryotes in sediments from the Changjiang Estuary and adjacent East China Sea. *Estuar. Coast. Shelf Sci.* 165 75–85. 10.1016/j.ecss.2015.09.005

[B24] Headd B. Engel A. S. (2013). Evidence for niche partitioning revealed by the distribution of sulfur oxidation genes collected from areas of a terrestrial sulfidic spring with differing geochemical conditions. *Appl. Environ. Microbiol.* 79 1171–1182. 10.1128/AEM.02812-12 23220955 PMC3568610

[B25] Hernández-Landa R. C. Acosta-González G. Núñez-Lara E. Arias-González J. E. (2015). Spatial distribution of surgeonfish and parrotfish in the north sector of the mesoamerican barrier reef system. *Mar. Ecol.* 36 432–446. 10.1111/maec.12152

[B26] Huh C. A. Su C. C. (1999). Sedimentation dynamics in the East China Sea elucidated from 210Pb, 137Cs and 239,240Pu. *Mar. Geol.* 160 183–196.

[B27] Imachi H. Sekiguchi Y. Kamagata Y. Loy A. Qiu Y. L. Hugenholtz P. (2006). Non-sulfate-reducing, syntrophic bacteria affiliated with *Desulfotomaculum* cluster I are widely distributed in methanogenic environments. *Appl. Environ. Microbiol.* 72 2080–2091. 10.1128/AEM.72.3.2080-2091.2006 16517657 PMC1393244

[B28] Jemaneh Z. Qiang S. Wang J. G. Huang M. Y. Xia F. Wu J. H. (2013). Effects of *Spartina alterniflora* invasion on the communities of methanogens and sulfate-reducing bacteria in estuarine marsh sediments. *Front. Microbiol.* 4:243. 10.3389/fmicb.2013.00243 23986751 PMC3750361

[B29] Jiang L. J. Zheng Y. P. Peng X. T. Zhou H. Y. Zhang C. L. Xiao X. (2009). Vertical distribution and diversity of sulfate-reducing prokaryotes in the Pearl River estuarine sediments, Southern China. *FEMS Microbiol. Ecol.* 70 249–262. 10.1111/j.1574-6941.2009.00758.x 19744241

[B30] Jiao N. Z. Yang Y. H. Hong N. Ma Y. Harada S. Koshikawa H. S. (2005). Dynamics of autotrophic picoplankton and heterotrophic bacteria in the East China Sea. *Cont. Shelf Res.* 25 1265–1279. 10.1016/j.csr.2005.01.002

[B31] Jørgensen B. B. (1982). Mineralization of organic matter in the sea bed-the role of sulphate reduction. *Nature* 296 643–645. 10.1038/296643a0

[B32] Jørgensen B. B. Bang M. Blackburn T. H. (1990). Anaerobic mineralization in marine sediments from the Baltic Sea–North Sea transition. *Mar. Ecol. Prog. Ser.* 59 39–54. 23825619

[B33] Jørgensen B. B. Nelson D. C. (2004). Sulfide oxidation in marine sediments: geochemistry meets microbiology. *Geol. Soc. Am. Spec.* 379 63–81. 10.1130/0-8137-2379-5.63

[B34] Jung J. S. Jang K. H. Sihn E. H. Park S. K. Park C. H. (2005). Characteristics of sulfur oxidation by a newly isolated *Burkholderia* spp. *J. Microbiol. Biotechnol.* 15 716–721.

[B35] Kjeldsen K. U. Loy A. Jakobsen T. F. Thomse T. R. Wagner M. Ingvorse K. (2007). Diversity of sulfate-reducing bacteria from an extreme hypersaline sediment, Great Salt Lake (Utah). *FEMS Microbiol. Ecol.* 60 287–298. 10.1111/j.1574-6941.2007.00288.x 17367515

[B36] Kojima H. Watanabe T. Iwata T. Fukui M. (2014). Identification of major planktonic sulfur oxidizers in stratified freshwater lake. *PLOS ONE* 9:e0093877. 10.1371/journal.pone.0093877 24695535 PMC3973623

[B37] Kondo R. Shigematsu K. Kawahara N. Okarmura T. Yoon Y. H. Sakami T. (2012). Abundance of sulphate-reducing bacteria in fish farm sediments along the coast of Japan and South Korea. *Fish. Sci.* 78 123–131. 10.1007/s12562-011-0439-3

[B38] Krishnani K. K. Kathiravan V. Natarajan M. Kailasam M. Pillai S. M. (2010). Diversity of sulfur-oxidizing bacteria in Greenwater system of coastal aquaculture. *Appl. Biochem. Biotechnol.* 162 1225–1237. 10.1007/s12010-009-8886-3 20069462

[B39] Leloup J. Fossing H. Kohls K. Holmkvist L. Borowski C. Jørgensen B. B. (2009). Sulfate-reducing bacteria in marine sediment (Aarhus Bay, Denmark): abundance and diversity related to geochemical zonation. *Environ. Microbiol.* 11 1278–1291. 10.1111/j.1462-2920.2008.01855.x 19220398

[B40] Lenk S. Arnds J. Zerjatke K. Musat N. Amann R. Mußmann M. (2011). Novel groups of *Gammaproteobacteria* catalyse sulfur oxidation and carbon fixation in a coastal, intertidal sediment. *Environ. Microbiol.* 13 758–774. 10.1111/j.1462-2920.2010.02380.x 21134098

[B41] Liu J. P. Li A. C. Xu K. H. Velozzi D. M. Yang Z. S. Milliman J. D. (2006). Sedimentary features of the Yangtze river-derived along-shelf clinoform deposit in the East China Sea. *Cont. Shelf Res.* 26 2141–2156. 10.1016/j.csr.2006.07.013

[B42] Loy A. Duller S. Baranyi C. Mußmann M. Ott J. Sharon I. (2009). Reverse dissimilatory sulfite reductase as phylogenetic marker for a subgroup of sulfur-oxidizing prokaryotes. *Environ. Microbiol.* 11 289–299. 10.1111/j.1462-2920.2008.01760.x 18826437 PMC2702494

[B43] Luo J. F. Lin W. T. Guo Y. (2011). Functional genes based analysis of sulfur-oxidizing bacteria community in sulfide removing bioreactor. *Appl. Microbiol. Biotechnol.* 90 769–778. 10.1007/s00253-010-3061-x 21212946

[B44] Magoè T. Salzberg S. L. (2011). FLASH: fast length adjustment of short reads to improve genome assemblies. *Bioinformatics* 27 2957–2963. 10.1093/bioinformatics/btr507 21903629 PMC3198573

[B45] Mahmoudi N. Robeson M. S. Castro H. F. Fortney J. L. Techtmann S. M. Joyner D. C. (2015). Microbial community composition and diversity in Caspian Sea sediments. *FEMS Microbiol. Ecol.* 91 1–11. 10.1093/femsec/fiu013 25764536 PMC4399438

[B46] Masuda S. Bao Z. Okubo T. Sasaki K. Ikeda S. Shinoda R. (2016). Sulfur fertilization changes the community structure of rice root-, and soil- associated bacteria. *Microbes Environ.* 31 70–75. 10.1264/jsme2.ME15170 26947443 PMC4791119

[B47] Masuda S. Eda S. Ikeda S. Mitsui H. Minamisawa K. (2010). Thiosulfate-dependent chemolithoautotrophic growth of *Bradyrhizobium japonicum*. *Appl. Environ. Microbiol.* 76 2402–2409. 10.1128/AEM.02783-09 20173070 PMC2849208

[B48] Meyer B. Imhoff J. F. Kuever J. (2007). Molecular analysis of the distribution and phylogeny of the *soxB* gene among sulfur-oxidizing bacteria-evolution of the Sox sulfur oxidation enzyme system. *Environ. Microbiol.* 9 2957–2977. 10.1111/j.1462-2920.2007.01407.x 17991026

[B49] Meyer B. Kuever J. (2007). Molecular analysis of the diversity of sulfate-reducing and sulfur-oxidizing prokaryotes in the environment, using *aprA* as functional marker gene. *Appl. Environ. Microbiol.* 73 7664–7679. 10.1128/AEM.01272-07 17921272 PMC2168068

[B50] Meyer D. D. de Andrade P. A. M. Durrer A. Andreote F. D. Corção G. Brandelli A. (2016). Bacterial communities involved in sulfur transformations in wastewater treatment plants. *Appl. Microbiol. Biotechnol.* 100 10125–10135. 10.1007/s00253-016-7839-3 27683212

[B51] Michelsen C. F. Pedas P. Glaring M. A. Schjoerring J. K. Stougaard P. (2014). Bacterial diversity in Greenlandic soils as affected by potato cropping and inorganic versus organic fertilization. *Polar Biol.* 37 61–71. 10.1007/s00300-013-1410-9

[B52] Milliman J. D. Meade R. H. (1983). World-wide delivery of river sediment to the oceans. *J. Geol.* 91 1–21.

[B53] Miyashita A. Mochimaru H. Kazama H. Ohashi A. Yamaguchi T. Nunoura T. (2009). Development of 16s rRNA gene-targeted primers for detection of archaeal anaerobic methanotrophs (ANMEs). *FEMS Microbiol. Lett.* 297 31–37. 10.1111/j.1574-6968.2009.01648.x 19486160

[B54] Müller A. L. Kjeldsen K. U. Rattei T. Pester M. Loy A. (2014). Phylogenetic and environmental diversity of *DsrAB*-type dissimilatory (bi) sulfite reductases. *ISME J.* 9 1152–1165. 10.1038/ismej.2014.208 25343514 PMC4351914

[B55] Nie M. Wang M. Li B. (2009). Effects of salt marsh invasion by Spartina alterniflora on sulfate-reducing bacteria in the Yangtze River estuary, China. *Ecol. Eng.* 35 1804–1808. 10.1016/j.ecoleng.2009.08.002 23986751

[B56] Oksanen J. Blanchet F. G. Kindt R. Legengre P. Minchin P. R. O’Hara R. B. (2011). *Vegan: Community Ecology Package. R Package Version 2.0-2*. Available at: http://CRAN.R-project.org/package=vegan

[B57] Pester M. Knorr K. H. Friedrich M. W. Wagner M. Loy A. (2012). Sulfate-reducing microorganisms in wetlands–fameless actors in carbon cycling and climate change. *Front. Microbiol.* 3:72 10.3389/fmicb.2012.00072 PMC3289269 22403575

[B58] Petri R. Podgorsek L. Imhoff J. F. (2001). Phylogeny and distribution of the *soxB* gene among thiosulfate-oxidizing bacteria. *FEMS Microbiol. Lett.* 197 171–178. 10.1016/S0378-1097(01)00111-2 11313131

[B59] Pham V. H. Yong J. J. Park S. J. Yoon D. N. Chung W. H. Rhee S. K. (2008). Molecular analysis of the diversity of the sulfide: quinone reductase (*sqr*) gene in sediment environments. *Microbiology* 154 3112–3121. 10.1099/mic.0.2008/018580-0 18832317

[B60] Purcell A. M. Mikucki J. A. Achberger A. M. Alekhina I. A. Barbante C. Christner B. C. (2014). Microbial sulfur transformations in sediments from Subglacial Lake Whillans. *Front. Microbiol.* 5:594. 10.3389/fmicb.2014.00594 25477865 PMC4237127

[B61] Quillet L. Besaury L. Popova M. Paissé S. Deloffre J. Ouddane B. (2012). Abundance, diversity and activity of sulfate-reducing prokaryotes in heavy metal-contaminated sediment from a salt marsh in the Medway Estuary (UK). *Mar. Biotechnol.* 14 363–381. 10.1007/s10126-011-9420-5 22124626

[B62] Reed H. E. Martiny J. B. H. (2013). Microbial composition affects the functioning of estuarine sediments. *ISME J.* 7 868–879. 10.1038/ismej.2012.154 23235294 PMC3603390

[B63] Saarenheimo J. Tiirola M. A. Rissanen A. J. (2015). Functional gene pyrosequencing reveals core proteobacterial denitrifiers in boreal lakes. *Front. Microbiol.* 6:674. 10.3389/fmicb.2015.00674 26191058 PMC4486872

[B64] Shen L. Xu H. Guo X. Meng L. (2011). Characteristics of large-scale harmful algal blooms (HABs) in the Yangtze River Estuary and the adjacent East China Sea (ECS) from 2000 to 2010. *J. Environ. Prot.* 2 1285–1294. 10.4236/jep.2011.210148

[B65] Sievert S. M. Hügler M. Taylor C. D. Wirsen C. O. (2008). “Sulfur oxidation at deep-sea hydrothermal vents,” in *Microbial Sulfur Metabolism* eds Dahl C. Friedrich C. G. (Berlin: Springer) 238–258. 10.1007/978-3-540-72682-1_19

[B66] Su C. C. Huh C. A. (2002). 210Pb, 137Cs and 239,240Pu in East China Sea sediments: sources, pathways and budgets of sediments and radionuclides. *Mar. Geol.* 183 163–178.

[B67] Tago K. Okubo T. Shimomura Y. Kikuchi Y. Hori T. Nagayama A. (2014). Environmental factors shaping the community structure of ammonia-oxidizing bacteria and archaea in sugarcane field soil. *Microbes Environ.* 30 21–28. 10.1264/jsme2.ME14137 25736866 PMC4356460

[B68] Thomas F. Giblin A. E. Cardon Z. G. Sievert S. M. (2014). Rhizosphere heterogeneity shapes abundance and activity of sulfur-oxidizing bacteria in vegetated salt marsh sediments. *Front. Microbiol.* 5:309. 10.3389/fmicb.2014.00309 25009538 PMC4068000

[B69] Tourna M. Maclean P. Condron L. O’Callaghan M. Wakelin S. A. (2014). Links between sulphur oxidation and sulphur-oxidising bacteria abundance and diversity in soil microcosms based on *soxB* functional gene analysis. *FEMS Microbiol. Ecol.* 88 538–549. 10.1111/1574-6941.12323 24646185

[B70] Treude T. Krause S. Maltby J. Dale A. W. Coffin R. Hamdan L. J. (2014). Sulfate reduction and methane oxidation activity below the sulfate-methane transition zone in Alaskan Beaufort Sea continental margin sediments: implications for deep sulfur cycling. *Geochim. Cosmochim. Acta* 144 217–237. 10.1016/j.gca.2014.08.018

[B71] Varon-Lopez M. Dias A. C. F. Fasanella C. C. Durrer A. Melo I. S. (2013). Sulphur-oxidizing and sulphate-reducing communities in Brazilian mangrove sediments. *Environ. Microbiol.* 16 845–855. 10.1111/1462-2920.12237 24033859

[B72] Wang K. Ye X. S. Zhang H. J. Chen H. P. Zhang D. M. Liu L. (2016). Regional variations in the diversity and predicted metabolic potential of benthic prokaryotes in coastal northern Zhejiang, East China Sea. *Sci. Rep.* 6:38709. 10.1038/srep38709 27917954 PMC5137025

[B73] Wang L. P. Zheng B. H. Lei K. (2015). Diversity and distribution of bacterial community in the coastal sediments of Bohai Bay, China. *Acta Oceanol. Sin.* 34 122–131. 10.1007/s13131-015-0719-3

[B74] Wu X. J. Pan J. L. Liu X. L. Tan J. Li D. T. Yang H. (2009). Sulfate-reducing bacteria in leachate-polluted aquifers along the shore of the East China Sea. *Can. J. Microbiol.* 55 818–828. 10.1139/W09-037 19767854

[B75] Xiong J. B. Ye X. S. Wang K. Chen H. P. Hu C. J. Zhu J. L. (2014). Biogeography of the sediment bacterial community responds to a nitrogen pollution gradient in the East China Sea. *Appl. Environ. Microbiol.* 80 1919–1925. 10.1128/AEM.03731-13 24413606 PMC3957648

[B76] Xu G. Liu J. Liu S. F. Wang Z. B. Hu G. Kong X. H. (2016). Modern muddy deposit along the Zhejiang coast in the East China Sea: response to large-scale human projects. *Cont. Shelf Res.* 130 68–78. 10.1016/j.csr.2016.10.007

[B77] Yang J. Jiang H. C. Dong H. L. Wu G. Hou W. G. Zhao W. Y. (2013). Abundance and diversity of sulfur-oxidizing bacteria along a salinity gradient in four Qinghai-Tibetan lakes, China. *Geomicrobiol. J.* 30 851–860. 10.1080/01490451.2013.790921

[B78] Yang X. Huang T. L. Guo L. Xia C. Zhang H. H. Zhou S. L. (2015). Abundance and diversity of sulfate-reducing bacteria in the sediment of the Zhou cun drinking water reservoir in Eastern China. *Genet. Mol. Res.* 14 5830–5844. 10.4238/2015.May.29.15 26125782

[B79] Ye Q. Wu Y. Zhu Z. Y. Wang X. N. Li Z. Q. Zhang J. (2016). Bacterial diversity in the surface sediments of the hypoxic zone near the Changjiang Estuary and in the East China Sea. *Microbiologyopen* 5 323–339. 10.1002/mbo3.330 26817579 PMC4831476

[B80] Yousuf B. Kumar R. Mishra A. Jha B. (2014). Unravelling the carbon and sulphur metabolism in coastal soil ecosystems using comparative cultivation-independent genome-level characterisation of microbial communities. *PLOS ONE* 9:e107025. 10.1317/journal.pone.0107025 25225969 PMC4167329

[B81] Yu Y. Song J. M. Li X. G. Yuan H. M. Li N. (2013). Fractionation, sources and budgets of potential harmful elements in surface sediments of the East China Sea. *Mar. Pollut. Bull.* 68 157–167. 10.1016/j.marpolbul.2012.11.043 23265773

[B82] Zhang W. Song L. S. Ki J. S. Lau C. K. Li X. D. Qian P. Y. (2008). Microbial diversity in polluted harbor sediments II: sulfate-reducing bacterial community assessment using terminal restriction fragment length polymorphism and clone library of *dsrAB* gene. *Estuar. Coast Shelf Sci.* 76 682–691. 10.1016/j.ecss.2007.07.039

[B83] Zhang Y. Xie X. Jiao N. Hsiao S. S. Y. Kao S. J. (2014). Diversity and distribution of *amoA*-type nitrifying and *nirS*-type denitrifying microbial communities in the Yangtze River estuary. *Biogeosciences* 10 17819–17857. 10.5194/bg-11-2131-2014

[B84] Zhang Y. Zhen Y. Mi T. Z. He H. Yu Z. G. (2016). Molecular characterization of sulfate-reducing bacteria community in surface sediments from the adjacent area of Changjiang Estuary. *J. Ocean Univ. China* 15 107–116. 10.1007/s11802-016-2781-7

[B85] Zhu D. C. Tanabe S. H. Yang C. Zhang W. M. Sun J. Z. (2013). Bacterial community composition of South China Sea sediments through pyrosequencing-based analysis of 16S rRNA genes. *PLOS ONE* 8:e78501. 10.1371/journal.pone.0078501 24205246 PMC3804488

