# Supplementary material for: Microbial Diversity and Community Structure of Sulfate-Reducing and Sulfur-Oxidizing Bacteria in Sediment Cores from the East China Sea
Source: Front Microbiol. 2017 Nov 7;8:2133. doi: 10.3389/fmicb.2017.02133 (PMC5682103; doi:10.3389/fmicb.2017.02133)
Supplement: Supplementary file 1 [file Presentation_1.DOCX]

Supplementary Material

Microbial Diversity and Community Structure of Sulfate-Reducing and Sulfur-Oxidizing Bacteria in Sediment Cores from the East China Sea

**Yu Zhang^1,2,3^, Xungong Wang^1,2,3^, Yu Zhen^1,2,3,*^, Tiezhu Mi^1,2,3^, Hui He^2,3,4^, Zhigang Yu^3,5^**

*** Correspondence:** Yu Zhen: zhenyu@ouc.edu.cn

# Supplementary Tables

**Table S1.** Environmental parameters of the sediment pore-water at the sampling stations

| Station | Depth  (cm) | Fe(II)  (µM) | Mn(II)  (µM) | SO_4_-S  (mM) | Depth  (cm) | pH | NH_4_-N  (µM) | NO_2_-N  (µM) | NO_3_-N  (µM) | DIN^a^  (µM) | PO_4_-P  (µM) | SiO_3_-Si  (µM) |
| --- | --- | --- | --- | --- | --- | --- | --- | --- | --- | --- | --- | --- |
| S31 | 0 | 6.71 | 102.11 | 26.98 | 0-1 | 7.47 | 88.80 | 0.08 | 2.55 | 91.44 | 3.44 | 125.35 |
|  | 1 | 9.41 | 103.82 | 26.64 | 1-2 | 7.52 | 156.17 | 0.02 | 3.26 | 159.45 | 3.26 | 116.74 |
|  | 2 | 9.51 | 129.56 | 26.67 | 2-3 | 7.64 | 90.82 | - | 2.04 | 92.86 | 2.31 | 104.60 |
|  | 3 | 6.17 | 171.43 | 26.31 | 3-4 | 7.43 | 223.90 | 0.03 | 2.94 | 226.88 | 3.33 | 134.14 |
|  | 4 | 56.15 | 150.75 | 25.79 | 4-5 | 7.32 | 306.30 | 0.02 | 2.48 | 308.80 | 7.46 | 215.95 |
|  | 5 | 7.52 | 192.17 | 25.73 | 5-6 | 7.33 | 366.88 | 0.08 | 2.30 | 369.26 | 5.16 | 233.81 |
|  | 6 | 39.2 | 82.68 | 25.74 | 6-7 | 7.33 | 379.63 | - | 1.78 | 381.41 | 6.74 | 232.70 |
|  | 7 | 110.5 | 137.51 | 25.50 | 7-8 | 7.29 | 383.55 | - | 2.11 | 385.66 | 9.26 | 257.21 |
|  | 8 | 133.17 | 139.17 | 24.67 | 8-9 | 7.29 | 414.98 | - | 2.84 | 417.82 | 12.17 | 285.55 |
|  | 9 | 151.47 | 100.09 | 24.19 | 9-10 | 7.34 | 395.08 | - | 2.14 | 397.22 | 9.00 | 289.35 |
|  | 10 | 95.67 | 66.99 | 26.56 | 10-12 | 7.34 | 447.25 | 0.03 | 1.93 | 449.21 | 9.44 | 300.18 |
|  | 12 | 63.9 | 34.73 | 26.98 | 12-14 | 7.38 | 398.90 | 0.00 | 2.13 | 401.03 | 9.24 | 248.14 |
|  | 14 | 39.29 | 19.95 | 25.85 | 14-16 | 7.42 | 338.37 | - | 2.38 | 340.75 | 5.98 | 217.75 |
|  | 16 | 39.2 | 15.99 | 26.06 | 16-18 | 7.45 | 323.24 | 0.14 | 2.40 | 325.79 | 5.65 | 197.30 |
|  | 18 | 15.54 | 6.69 | 25.80 | 18-20 | 7.46 | 346.54 | 0.10 | 2.31 | 348.95 | 5.28 | 208.09 |
|  | 20 | 14.74 | 8.12 | 25.59 | 20-22 | 7.41 | 261.05 | 0.00 | 4.23 | 265.28 | 5.16 | 156.26 |
| S33 | 0 | 5.57 | 31.46 | 24.62 | 0-1 | 8.41 | 12.72 | 0.25 | 5.84 | 18.81 | 8.83 | 225.66 |
|  | 1 | 34.09 | 59.92 | 25.39 | 1-2 | 8.63 | 45.11 | 0.47 | 4.42 | 49.99 | 11.14 | 289.04 |
|  | 2 | 52.48 | 41.85 | 24.75 | 2-3 | 8.64 | 146.33 | 0.09 | 1.63 | 148.05 | 11.90 | 282.04 |
|  | 3 | 33.19 | 52.12 | 24.11 | 3-4 | 8.64 | 106.20 | 0.07 | 1.59 | 107.87 | 11.27 | 278.99 |
|  | 4 | 61.7 | 48.84 | 24.45 | 4-5 | 8.54 | 92.05 | 0.08 | 2.21 | 94.34 | 13.06 | 288.48 |
|  | 5 | 50.38 | 44.19 | 25.11 | 5-6 | 8.54 | 89.95 | 0.13 | 2.23 | 92.31 | 10.33 | 254.99 |
|  | 6 | 51.25 | 37.25 | 24.12 | 6-7 | 8.49 | 84.95 | 0.08 | 2.32 | 87.35 | 11.27 | 260.55 |
|  | 7 | 43.96 | 40.3 | 25.46 | 7-8 | 8.48 | 121.51 | 0.15 | 1.75 | 123.41 | 11.84 | 284.62 |
|  | 8 | 59.23 | 43.21 | 25.18 | 8-9 | 8.52 | 131.84 | 0.06 | 1.60 | 133.50 | 12.51 | 337.35 |
|  | 9 | 50.35 | 41.14 | 24.87 | 9-10 | 8.52 | 106.15 | 0.13 | 1.70 | 107.97 | 12.15 | 291.62 |
|  | 10 | 24.84 | 34.47 | 24.95 | 10-12 | 8.49 | 111.24 | 0.06 | 1.82 | 113.12 | 11.07 | 261.59 |
|  | 12 | 28.27 | 39.66 | 25.33 | 12-14 | 8.48 | 118.56 | 0.03 | 2.13 | 120.72 | 11.16 | 258.72 |
|  | 14 | 32.62 | 28.67 | 25.27 | 14-16 | 8.50 | 101.03 | 0.11 | 2.57 | 103.70 | 9.27 | 243.39 |
|  | 16 | 27.42 | 31.53 | 25.87 | 16-18 | 8.51 | 116.78 | 0.04 | 2.94 | 119.76 | 9.66 | 246.28 |
|  | 18 | 13.23 | 50.15 | 26.01 | 18-20 | 8.52 | 135.78 | 0.02 | 2.25 | 138.05 | 11.11 | 283.38 |
|  | 20 | 31.82 | 27.38 | 25.88 | 20-22 | 8.54 | 105.86 | 0.08 | 2.17 | 108.11 | 9.46 | 254.36 |
|  | 22 | 34.23 | 22.35 | 25.65 | 22-24 | 8.53 | 130.91 | 0.12 | 1.92 | 132.95 | 8.61 | 243.69 |
|  | 24 | 45.82 | 48.37 | 24.86 | 24-26 | 8.52 | 141.28 | 0.11 | 1.53 | 142.92 | 8.80 | 239.83 |
|  | 26 | 28.57 | 23.35 | 25.54 | 26-28 | 8.50 | 151.65 | 0.10 | 1.13 | 152.89 | 8.99 | 244.69 |
|  | 28 | 19.73 | 20.9 | 26.18 | 28-30 | 8.52 | 132.94 | 0.05 | 1.73 | 134.72 | 9.54 | 210.16 |
|  | 30 | 25.36 | 23.11 | 25.08 | 30-32 | 8.56 | 132.95 | 0.30 | 1.58 | 134.84 | 8.56 | 239.28 |
|  | 32 | 18.71 | 20.36 | 26.13 | 32-34 | 8.59 | 116.09 | 0.08 | 4.56 | 120.73 | 9.86 | 262.90 |

a: dissolved inorganic nitrogen; “-”: undetected

**Table S2** Primers used in this study

| Target genes | Primer | Sequence (5′-3′) | Reference |
| --- | --- | --- | --- |
| 16S rRNA^a^ | 341F | CCT ACG GGA GGC AGC AG | Dang et al. (2010) |
|  | 518R | ATT ACC GCG GCT GCT GG |  |
| 16S rRNA^b^ | 341F | CCT AYG GGR BGC ASC AG | Michelsen et al.(2014) |
|  | 806R | GGA CTA CNN GGG TAT CTA AT |  |
| *dsrB*^a,b^ | DSRp2060F | CAA CAT CGT YCA YAC CCA GGG | Geets et al. (2006) |
|  | DSR4R | GTG TAG CAG TTA CCG CA |  |
| *soxB*^a^ | soxB693F | ATCGGNCARGCNTTYCCNTA | Krishnani et al. (2010) |
|  | soxB1164BK145 | AAGTTGCCDCGNCGRTA |  |
| *soxB*^b^ | soxB693F | ATC GGN CAR GCN TTY CCN TA | Meyer et al. (2007) |
|  | soxB1446B | CAT GTC NCC NCC RTG YTG |  |

a: genes used for qPCR analyses

b: genes used for high-throughput sequencing analyses

# Supplementary Figures





**Fig. S1** Sampling locations in the East China Sea

The shaded areas in the figure represent mud areas.





**Fig. S2** Vertical profiles of ^210^Pb_excess_ concentrations at S31 and S33
